# Supplementary material for: BRANE Cut: biologically-related a priori network enhancement with graph cuts for gene regulatory network inference
Source: BMC Bioinformatics. 2015 Nov 4;16:368. doi: 10.1186/s12859-015-0754-2 (PMC4634801; doi:10.1186/s12859-015-0754-2)
Supplement: Additional file 1 — BRANE Cut: Model parameters and sensitivity analysis. The document provides detailed justification for the choice of the model parameters, studies their relative influence and provides a sensitivity analysis. (PDF 114kb) [file 12859_2015_754_MOESM1_ESM.pdf]

Additional file 1

BRANE Cut: Model parameters and sensitivy analysis

Aurélie Pirayre, Camille Couprie, Frédérique Bidard, Laurent Duval, Jean-Christophe Pesquet

October 22, 2015

Contents

1 Choice of the lambda parameter **1**

2 Performance comparison of various methods on DREAM4 **2**

3 Contribution of each term of the Graph cut functional **3**

4 Heuristic and sensitivity analysis **6**

4.1 Heuristic on the μ parameter 6

4.2 Effects of the μ parameter 6

4.3 Additional validation of heuristics on DREAM5 7

1 Choice of the lambda parameter

To study the impact of the parameter λ_{TF} on the results, we display in Figure 1 Precision-Recall curves using the different possible combinations of $\lambda_{\overline{\mathrm{TF}}}$ and λ_{TF} on the DREAM4 dataset.

More specifically, we used the GENIE3 weights as an input to BRANE Cut. Variables μ and γ are set to 3 and the $(G-1)^{\mathrm{th}}$ quantile of the normalized weights, respectively. The Precision-Recall curve is computed by varying the $\lambda_{\overline{\mathrm{TF}}}$ parameter in a limited interval $[0, 0.1]$ to be able to plot a full Precision-Recall curve. For each $\lambda_{\overline{\mathrm{TF}}}$, we vary the λ_{TF} parameter with a linear sampling between the actual $\lambda_{\overline{\mathrm{TF}}}$ value and 1.

The results in terms of Area Under the Precision-Recall curves are provided in Table 1.

Network Index	GENIE3	BC-GENIE3	Gain vs GENIE3
1	0.269	**0.284**	5.57 %
2	0.288	**0.312**	8.33 %
3	0.331	**0.366**	10.57 %
4	0.323	**0.339**	4.95 %
5	0.329	**0.351**	6.69 %

Table 1: Area Under the Precision-Recall curves obtained for both GENIE3 and BRANE Cut methods on the five networks of the DREAM4 dataset. Here, BRANE Cut is initialized using GENIE3 weights. Relative AUPR gains are also provided.

Many different choices of $\lambda_{\overline{\mathrm{TF}}} \leq \lambda_{\mathrm{TF}}$ are possible These results show that such an extended choice for λ_{TF} is not critical to improve the performance over GENIE3. This validates our choice to reduce the initial number of thresholds to only one, similarly to the compared methods, as explained in the paper.

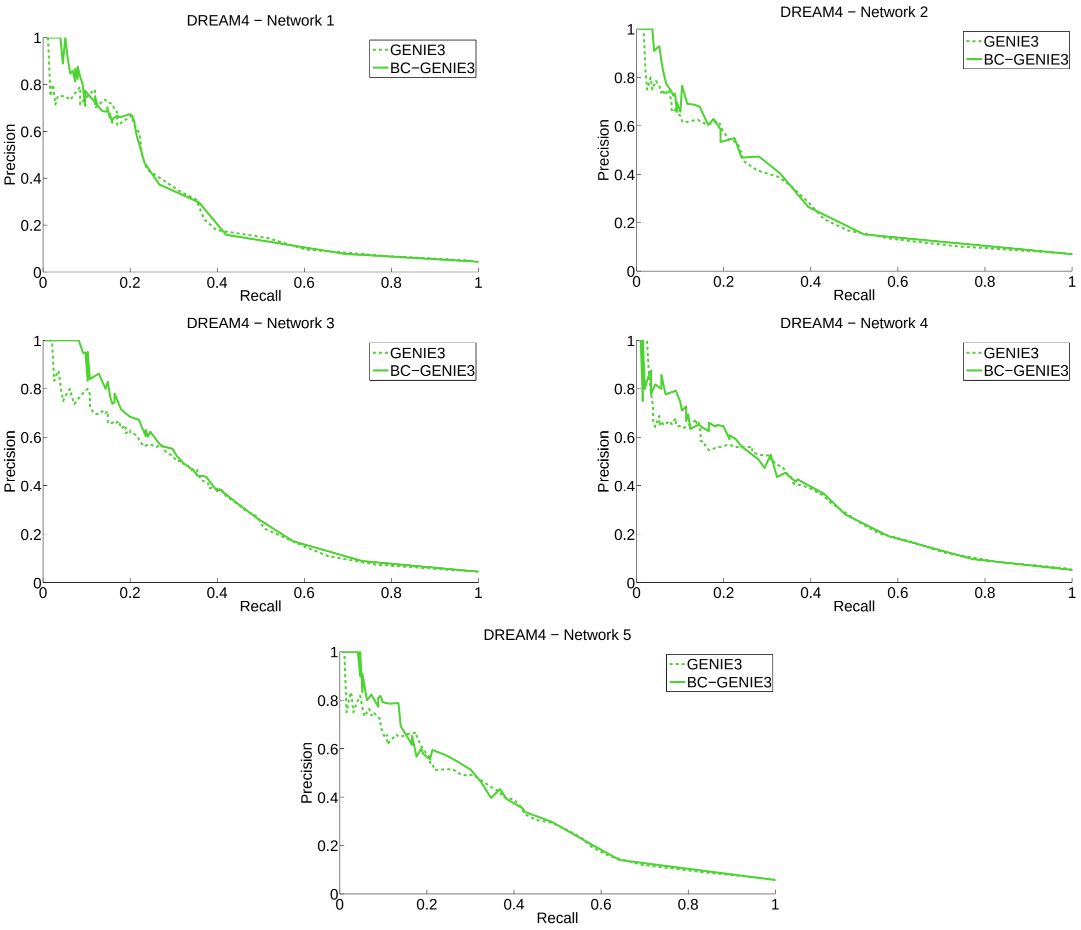

Figure 1: Precision-Recall curves obtained for both GENIE3 and BRANE Cut methods on the five networks of the DREAM4 dataset. Here, BRANE Cut is initialized using GENIE3 weights.

2 Performance comparison of various methods on DREAM4

In this section, we present detailed results obtained using BRANE Cut with CLR, GENIE3, ND-CLR or ND-GENIE3 weights.

Figures 2 to 6 show the resulting Precision-Recall curves. They were obtained by varying only the $\lambda_{\overline{\mathrm{TF}}}$ threshold as specified in the paper.

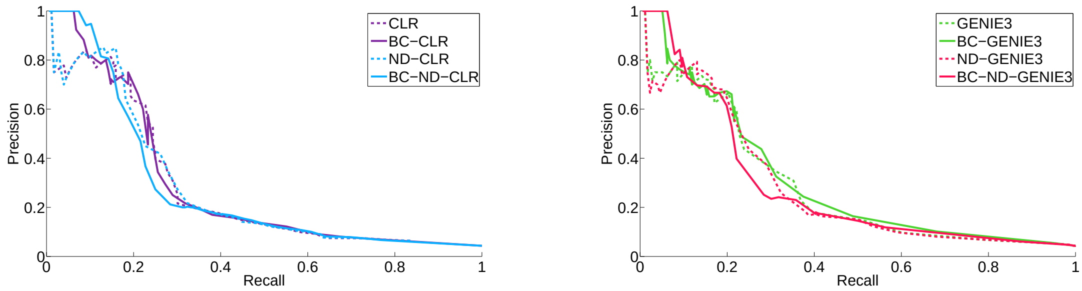

(a) Based on CLR weights (b) Based on GENIE3 weights

Figure 2: PR curves for CLR, GENIE3, ND-CLR, ND-GENIE3 and their BRANE Cut post-processing on Network 1 from the DREAM4 multifactorial challenge.

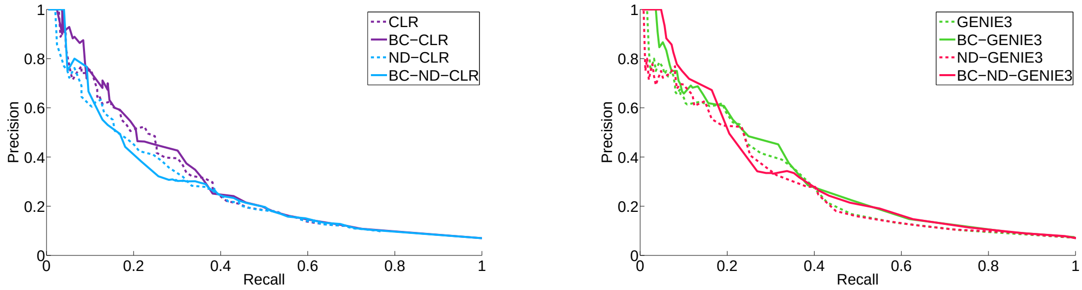

(a) Based on CLR weights (b) Based on GENIE3 weights

Figure 3: PR curves for CLR, GENIE3, ND-CLR, ND-GENIE3 and their BRANE Cut post-processing on Network 2 from the DREAM4 multifactorial challenge.

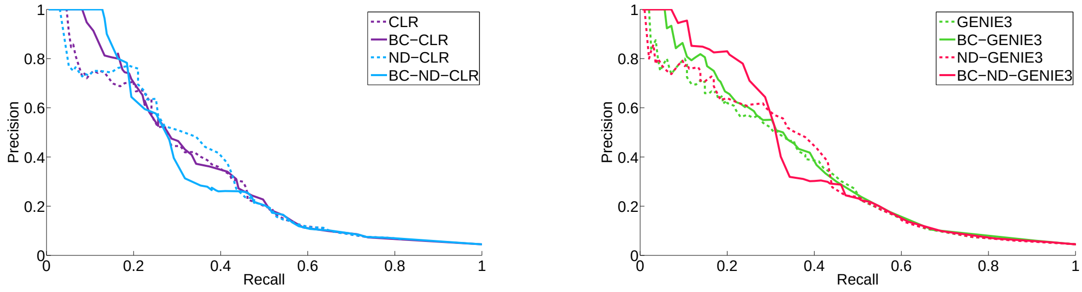

(a) Based on CLR weights (b) Based on GENIE3 weights

Figure 4: PR curves for CLR, GENIE3, ND-CLR, ND-GENIE3 and their BRANE Cut post-processing on Network 3 from the DREAM4 multifactorial challenge.

3 Contribution of each term of the Graph cut functional

To determine the influence of the different biological a priori introduced in our model (as expressed by Equation (2)), we present three Precision-Recall curves obtained using the *E. coli* dataset described in the

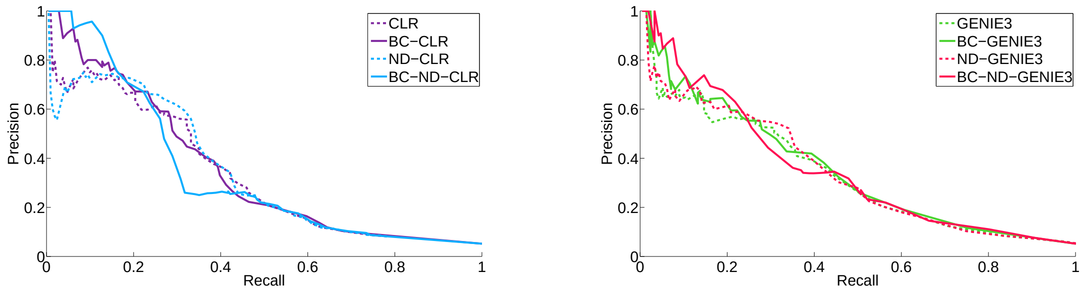

(a) Based on CLR weights (b) Based on GENIE3 weights

Figure 5: PR curves for CLR, GENIE3, ND-CLR, ND-GENIE3 and their BRANE Cut post-processing on Network 4 from the DREAM4 multifactorial challenge.

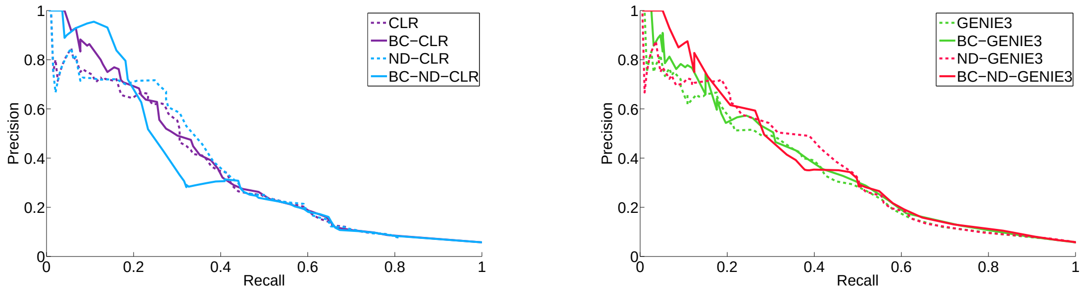

(a) Based on CLR weights (b) Based on GENIE3 weights

Figure 6: PR curves for CLR, GENIE3, ND-CLR, ND-GENIE3 and their BRANE Cut post-processing on Network 5 from the DREAM4 multifactorial challenge.

paper. The first PR curve is obtained without any a priori, the second one using the first a priori, and the third one using all of them. The curves are displayed in Figure 7.

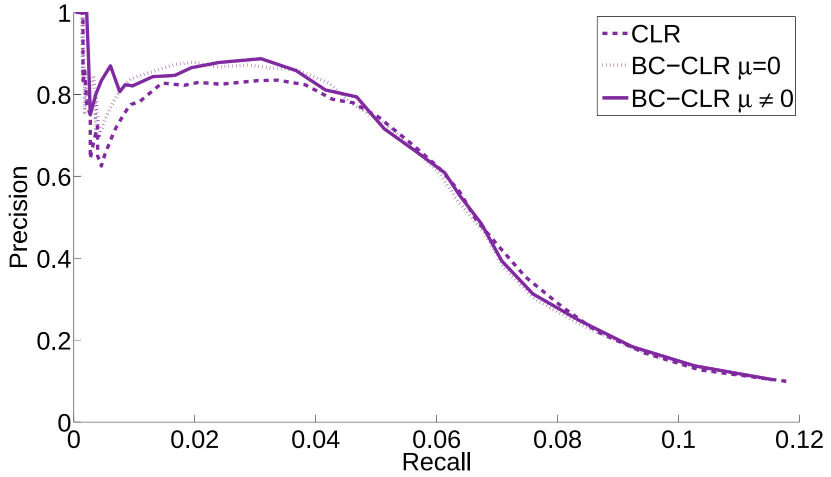

Figure 7: Precision Recall curve using BRANE Cut with different parameters μ and λ

The employed weights ω are those computed with CLR using the default parameters. We set $\lambda_{\text{TF}} = \beta\lambda_{\overline{\text{TF}}}$, $\beta \geq 1$ and incrementally assess the effectiveness of both β and μ choices.

The first curve is obtained using $\beta = 1$, and $\mu = 0$. These parameters make our method (thus, without a priori) equivalent to CLR, with $\lambda = \lambda_{\overline{\text{TF}}}$ acting as a unique threshold on ω weights. The second curve is obtained with $\mu = 0$ and $\beta = \frac{|\mathcal{V}|}{|\mathcal{T}|}$, as specified in the paper The third curve is obtained by taking full advantage of the capabilities offered by our model: $\beta = \frac{|\mathcal{V}|}{|\mathcal{T}|}$ and $\mu = 1000$. The corresponding AUPR (Area Under the Precision-Recall curve) are given in Table 2.

	CLR	BC-CLR $\mu = 0$	BC-CLR $\mu \neq 0$
AUPR	0.0786	0.0870	0.0879
Gain vs CLR	-	10.7 %	11.8 %

Table 2: AUPR for BRANE Cut used with different parameters μ and λ: $\mu = 0$ and $\lambda_{\text{TF}} = \lambda_{\overline{\text{TF}}}$ (equivalent to CLR), $\mu = 0$ and $\lambda_{\text{TF}} \geq \lambda_{\overline{\text{TF}}}$ (equivalent to treat the problem without the co-regulation property), and $\mu > 0$ and $\lambda_{\text{TF}} \geq \lambda_{\overline{\text{TF}}}$ (all the a priori are taken into account).

Following the procedure presented in Figure 4 of the paper, we also compare the AUPR computed for different parts of the whole PR curves. For each range of Precision values, the relative improvement is computed as: AUPR(BC-CLR)/AUPR(CLR) for BC-CLR with $\mu = 0$ (dotted purple line) and BC-CLR with $\mu \neq 0$ (solid purple line), see Figure 8.

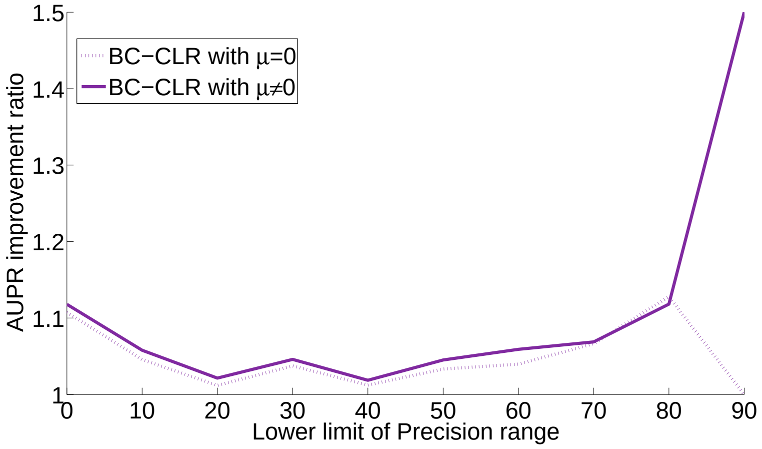

Figure 8: Improvements in terms of AUPR for different parts of the complete PR curves on the *Escherichia coli* network. In order to show the differential improvement over the Precision, relative AUPR are computed for different PR curves (for BC-CLR with $\mu = 0$ (dotted purple line) and BC-CLR with $\mu \neq 0$ (solid purple line)), truncated at different range of Precision: [0,100], [10,100], ...and [90,100]. Here, the improvement is defined as the AUPR ratio of BC-CLR and CLR.

4 Heuristic and sensitivity analysis

4.1 Heuristic on the μ parameter

Weights ω are employed to compute co-regulation probabilities $\rho_{i,j,j'}$. Different ω distributions lead to different sets of non-zero co-regulation probabilities. Consequently, they impact the optimal choice for μ. This is observed in the different μ values chosen for the tested networks. For practically useful inference, we consider important to obtain a simple estimation of μ for a given network. It should also be of low sensitivity. For a given set of weights, we denote by C_r the number of identified couples of genes $(j, j') \in \mathcal{T}^2$ co-regulating at least one gene. The total number of co-regulator couples, denoted by C_t, is equal to $\frac{|\mathcal{T}|(|\mathcal{T}|-1)}{2}$. We experimentally observe that an accurate order of magnitude close to the optimal μ is given by the cardinality-based ratio:

$$\mu = \frac{|\mathcal{T}|(|\mathcal{T}|-1)}{2C_r} \tag{1}$$

This heuristic is consistent with the biological view point, where a small proportion of co-regulator couples is expected. Results on DREAM4 using the proposed heuristic for the μ parameter are given in Table 3 and are consistent with whose presented in the article. In addition, the choice of this parameter allows us to obtain an AUPR of 0.0917 (resp. 0.0873) for BRANE Cut initialized with GENIE3 (resp. CLR) weights on the *E. coli* dataset.

Network index	1	2	3	4	5	Average
CLR	0.256	0.275	0.314	0.313	0.318	0.295
BC-CLR	**0.282**	0.309	0.344	0.343	**0.357**	0.327
GENIE3	0.269	0.288	0.331	0.323	0.329	0.308
BC-GENIE3	**0.297**	**0.320**	**0.356**	**0.344**	0.352	**0.334**
ND-CLR	0.254	0.250	0.324	0.318	0.331	0.295
BC-ND-CLR	0.274	0.278	0.334	0.334	0.343	0.313
ND-GENIE3	0.263	0.275	0.336	0.328	0.354	0.309
BC-ND-GENIE3	0.273	**0.312**	**0.367**	**0.346**	**0.368**	**0.333**

Table 3: Area Under Precision-Recall for CLR, GENIE3, ND-CLR, ND-GENIE3 and their BRANE Cut post-processing on the DREAM4 dataset. For each given network, the two maximal improvements are reported in bold.

4.2 Effects of the μ parameter

From the above observation, we analyze the impact of this parameter on the results in terms of AUPR. We perform this analysis on the DREAM4 dataset with the CLR and the GENIE3 weights as initial weights. We compute the mean and median AUPR, as well as the corresponding deviation measures (standard deviation and median absolute deviation), over 50 values between 0.1μ and 10μ, where μ is firstly computed using the proposed heuristic. Results in Table 4 show a low variability in the impact of parameter μ. We observe a difference regarding the standard and the median deviation, however the highest observed variability remains acceptable.

Network index	1	2	3	4	5
mean(AUPR)	0.279	0.308	0.342	0.344	0.355
var(AUPR)	0.0027	8.8e-4	7.4e-4	7.7e-4	8.3e-4
median(AUPR)	0.279	0.309	0.342	0.344	0.355
mad(AUPR)	0.0027	6.6e-4	5.7e-4	5.3e-4	5.4e-4

(a) Mean (resp. median) and standard deviation (resp. median absolute deviation) on the AUPR for various μ values with BRANE Cut initialized with CLR.

Network index	1	2	3	4	5
mean(AUPR)	0.296	0.321	0.356	0.344	0.351
var(AUPR)	7.4e-4	7.5e-4	3.3e-4	4.8e-4	9.2e-4
median(AUPR)	0.296	0.321	0.356	0.344	0.351
mad(AUPR)	5.8e-4	5.4e-4	1.86e-4	3.06e-4	7.2e-4

(b) Mean (resp. median) and standard deviation (resp. median absolute deviation) on the AUPR for various μ values with BRANE Cut initialized with GENIE3.

Table 4: Sensitivity analysis of the parameter μ on the five networks of DREAM4, using BRANE Cut with CLR and GENIE3 as initial weights.

4.3 Additional validation of heuristics on DREAM5

We evaluate BRANE Cut using both CLR and GENIE3 weights as input on the three networks of DREAM5 for which a ground truth is available. All the BRANE Cut parameters (β, γ, μ) are chosen using the proposed heuristics. The evaluation is performed using the same procedure as described in the paper. AUPR is reported in table 5.

Network index	1	3	4
CLR	0.252	0.035	0.008
BC-CLR	**0.270**	0.036	0.008
GENIE3	0.283	0.046	0.008
BC-GENIE3	**0.297**	0.047	0.008

Table 5: AUPR for CLR, GENIE3 and BRANE Cut using both pre-computed CLR and GENIE3 weights. Statistically significant improved results in terms of AUPR are given in bold.

For the first network, we observe an improvement over seven and five percent respectively for CLR and GENIE3. On the third network, the improvement over CLR and GENIE3 reaches 2.8 % and 2.1 %. Regarding the fourth network, the AUPR computed with every method is exceptionally low. As such, the relative AUPR differences are insignificant, within the numerical precision.

Results show that with the proposed heuristics, BRANE Cut significantly outperforms CLR and GENIE3 on the first network, and equals performance achieved by CLR and GENIE3 on the third and fourth networks.
